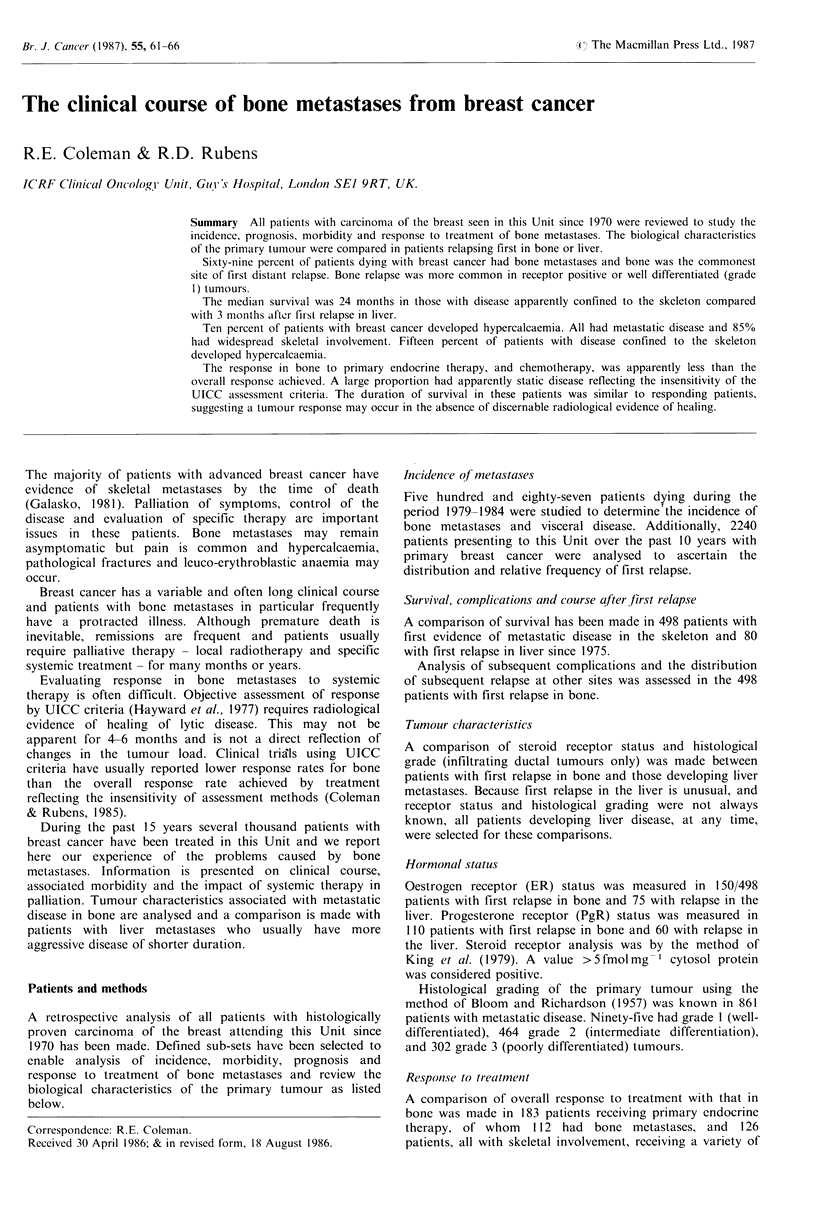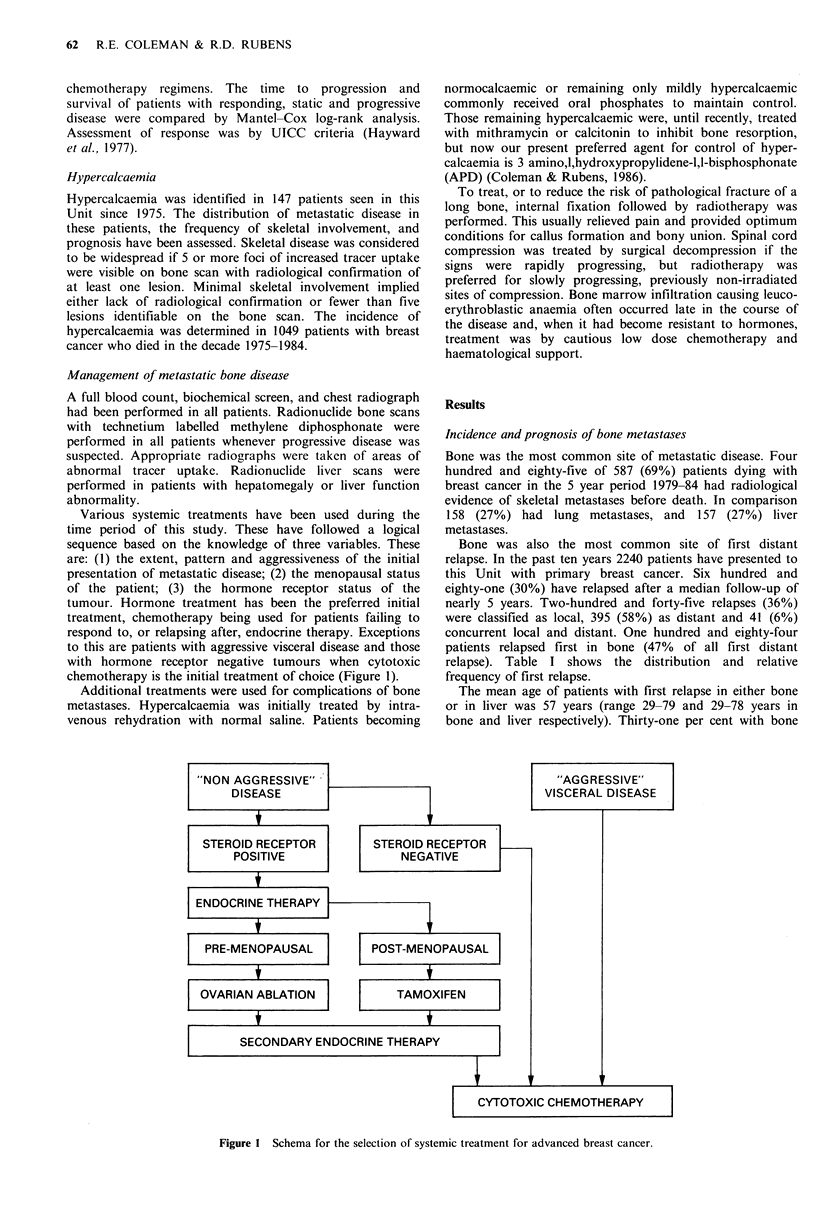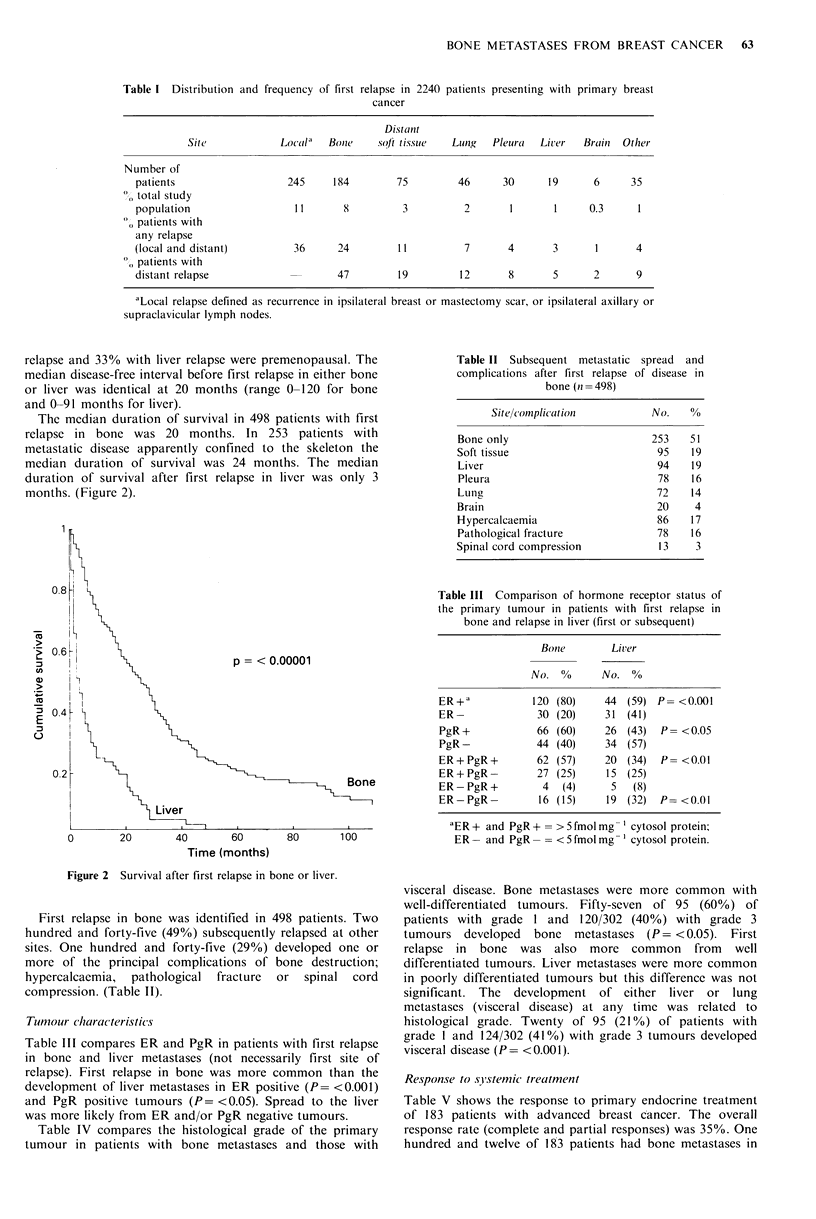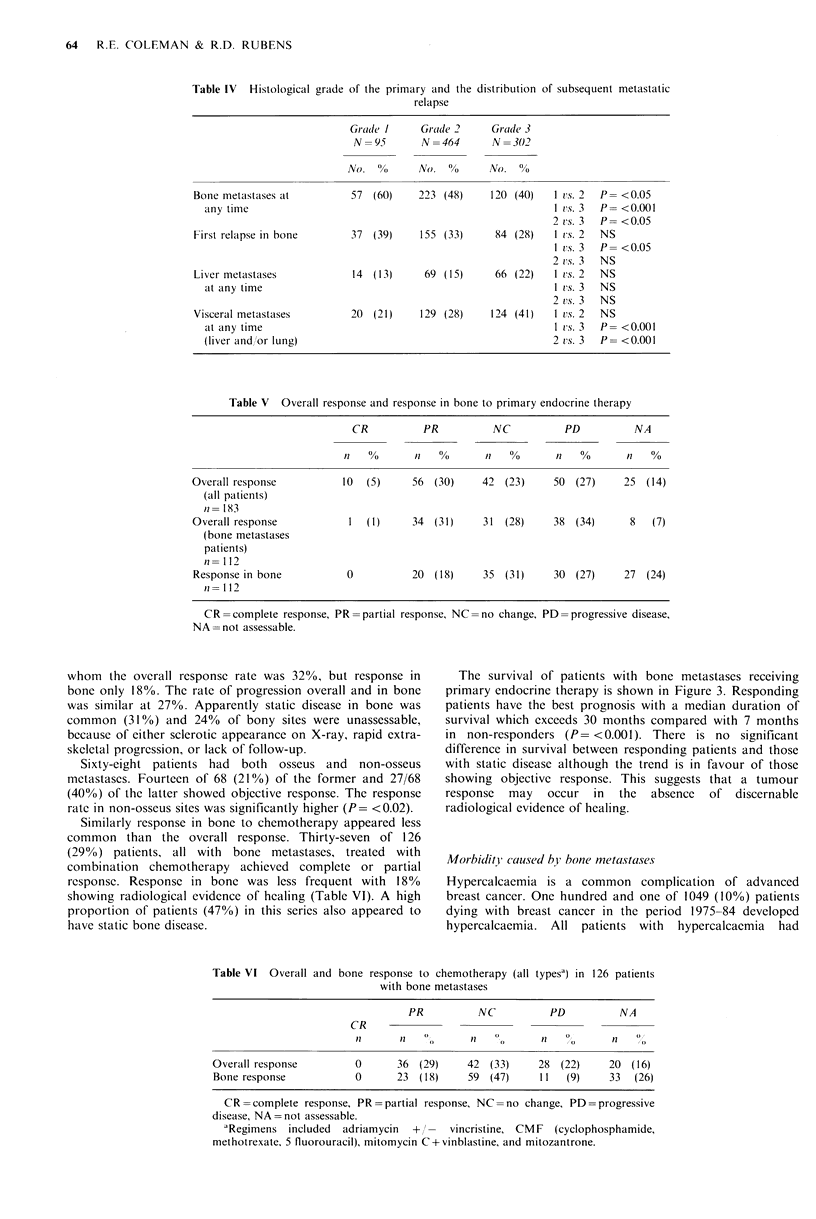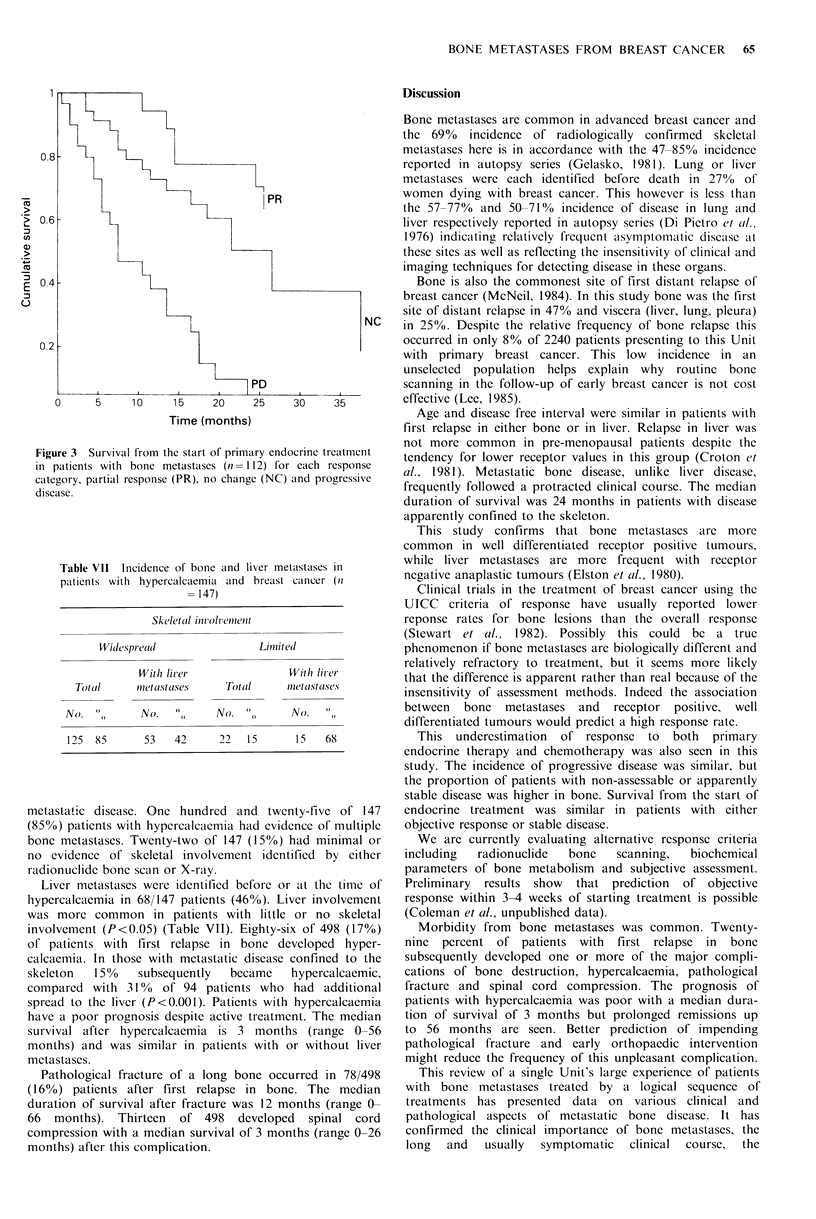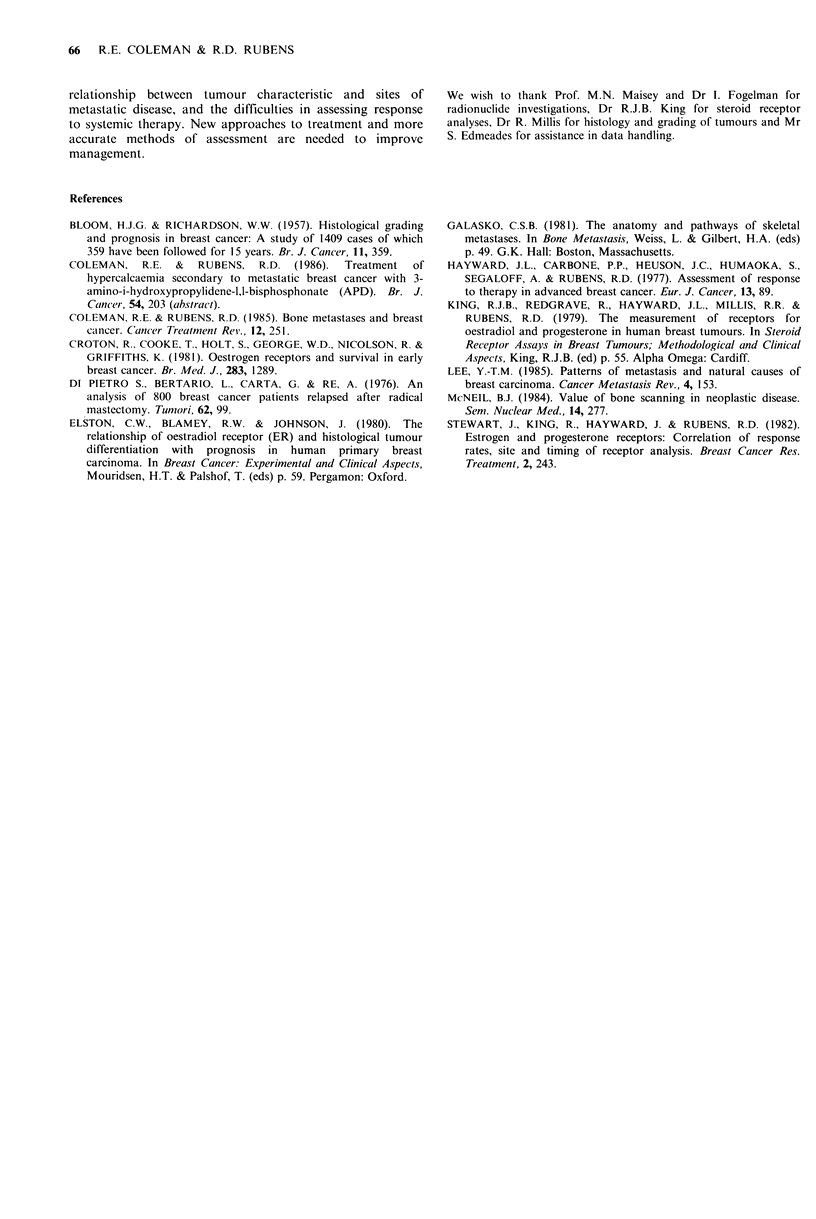# The clinical course of bone metastases from breast cancer.

**DOI:** 10.1038/bjc.1987.13

**Published:** 1987-01

**Authors:** R. E. Coleman, R. D. Rubens

## Abstract

All patients with carcinoma of the breast seen in this Unit since 1970 were reviewed to study the incidence, prognosis, morbidity and response to treatment of bone metastases. The biological characteristics of the primary tumour were compared in patients relapsing first in bone or liver. Sixty-nine percent of patients dying with breast cancer had bone metastases and bone was the commonest site of first distant relapse. Bone relapse was more common in receptor positive or well differentiated (grade 1) tumours. The median survival was 24 months in those with disease apparently confined to the skeleton compared with 3 months after first relapse in liver. Ten percent of patients with breast cancer developed hypercalcaemia. All had metastatic disease and 85% had widespread skeletal involvement. Fifteen percent of patients with disease confined to the skeleton developed hypercalcaemia. The response in bone to primary endocrine therapy, and chemotherapy, was apparently less than the overall response achieved. A large proportion had apparently static disease reflecting the insensitivity of the UICC assessment criteria. The duration of survival in these patients was similar to responding patients, suggesting a tumour response may occur in the absence of discernable radiological evidence of healing.


*Br. J. Cancer* (1987), **55**, 61–66 © The Macmillan Press Ltd., 1987

### The clinical course of bone metastases from breast cancer

R.E. Coleman & R.D. Rubens

*ICRF Clinical Oncology Unit, Guy's Hospital, London SE1 9RT, UK.*

**Summary** All patients with carcinoma of the breast seen in this Unit since 1970 were reviewed to study the incidence, prognosis, morbidity and response to treatment of bone metastases. The biological characteristics of the primary tumour were compared in patients relapsing first in bone or liver.

Sixty-nine percent of patients dying with breast cancer had bone metastases and bone was the commonest site of first distant relapse. Bone relapse was more common in receptor positive or well differentiated (grade 1) tumours.

The median survival was 24 months in those with disease apparently confined to the skeleton compared with 3 months after first relapse in liver.

Ten percent of patients with breast cancer developed hypercalcaemia. All had metastatic disease and 85% had widespread skeletal involvement. Fifteen percent of patients with disease confined to the skeleton developed hypercalcaemia.

The response in bone to primary endocrine therapy, and chemotherapy, was apparently less than the overall response achieved. A large proportion had apparently static disease reflecting the insensitivity of the UICC assessment criteria. The duration of survival in these patients was similar to responding patients, suggesting a tumour response may occur in the absence of discernable radiological evidence of healing.

The majority of patients with advanced breast cancer have evidence of skeletal metastases by the time of death (Galasko, 1981). Palliation of symptoms, control of the disease and evaluation of specific therapy are important issues in these patients. Bone metastases may remain asymptomatic but pain is common and hypercalcaemia, pathological fractures and leuco-erythroblastic anaemia may occur.

Breast cancer has a variable and often long clinical course and patients with bone metastases in particular frequently have a protracted illness. Although premature death is inevitable, remissions are frequent and patients usually require palliative therapy – local radiotherapy and specific systemic treatment – for many months or years.

Evaluating response in bone metastases to systemic therapy is often difficult. Objective assessment of response by UICC criteria (Hayward *et al.*, 1977) requires radiological evidence of healing of lytic disease. This may not be apparent for 4–6 months and is not a direct reflection of changes in the tumour load. Clinical trials using UICC criteria have usually reported lower response rates for bone than the overall response rate achieved by treatment reflecting the insensitivity of assessment methods (Coleman & Rubens, 1985).

During the past 15 years several thousand patients with breast cancer have been treated in this Unit and we report here our experience of the problems caused by bone metastases. Information is presented on clinical course, associated morbidity and the impact of systemic therapy in palliation. Tumour characteristics associated with metastatic disease in bone are analysed and a comparison is made with patients with liver metastases who usually have more aggressive disease of shorter duration.

### Patients and methods

A retrospective analysis of all patients with histologically proven carcinoma of the breast attending this Unit since 1970 has been made. Defined sub-sets have been selected to enable analysis of incidence, morbidity, prognosis and response to treatment of bone metastases and review the biological characteristics of the primary tumour as listed below.

Correspondence: R.E. Coleman.
Received 30 April 1986; & in revised form, 18 August 1986.

*Incidence of metastases*

Five hundred and eighty-seven patients dying during the period 1979–1984 were studied to determine the incidence of bone metastases and visceral disease. Additionally, 2240 patients presenting to this Unit over the past 10 years with primary breast cancer were analysed to ascertain the distribution and relative frequency of first relapse.

*Survival, complications and course after first relapse*

A comparison of survival has been made in 498 patients with first evidence of metastatic disease in the skeleton and 80 with first relapse in liver since 1975.

Analysis of subsequent complications and the distribution of subsequent relapse at other sites was assessed in the 498 patients with first relapse in bone.

*Tumour characteristics*

A comparison of steroid receptor status and histological grade (infiltrating ductal tumours only) was made between patients with first relapse in bone and those developing liver metastases. Because first relapse in the liver is unusual, and receptor status and histological grading were not always known, all patients developing liver disease, at any time, were selected for these comparisons.

*Hormonal status*

Oestrogen receptor (ER) status was measured in 150/498 patients with first relapse in bone and 75 with relapse in the liver. Progesterone receptor (PgR) status was measured in 110 patients with first relapse in bone and 60 with relapse in the liver. Steroid receptor analysis was by the method of King *et al.* (1979). A value $>5\,\text{fmol}\,\text{mg}^{-1}$ cytosol protein was considered positive.

Histological grading of the primary tumour using the method of Bloom and Richardson (1957) was known in 861 patients with metastatic disease. Ninety-five had grade 1 (well-differentiated), 464 grade 2 (intermediate differentiation), and 302 grade 3 (poorly differentiated) tumours.

*Response to treatment*

A comparison of overall response to treatment with that in bone was made in 183 patients receiving primary endocrine therapy, of whom 112 had bone metastases, and 126 patients, all with skeletal involvement, receiving a variety of chemotherapy regimens. The time to progression and survival of patients with responding, static and progressive disease were compared by Mantel–Cox log-rank analysis. Assessment of response was by UICC criteria (Hayward *et al.*, 1977).

*Hypercalcaemia*

Hypercalcaemia was identified in 147 patients seen in this Unit since 1975. The distribution of metastatic disease in these patients, the frequency of skeletal involvement, and prognosis have been assessed. Skeletal disease was considered to be widespread if 5 or more foci of increased tracer uptake were visible on bone scan with radiological confirmation of at least one lesion. Minimal skeletal involvement implied either lack of radiological confirmation or fewer than five lesions identifiable on the bone scan. The incidence of hypercalcaemia was determined in 1049 patients with breast cancer who died in the decade 1975–1984.

*Management of metastatic bone disease*

A full blood count, biochemical screen, and chest radiograph had been performed in all patients. Radionuclide bone scans with technetium labelled methylene diphosphonate were performed in all patients whenever progressive disease was suspected. Appropriate radiographs were taken of areas of abnormal tracer uptake. Radionuclide liver scans were performed in patients with hepatomegaly or liver function abnormality.

Various systemic treatments have been used during the time period of this study. These have followed a logical sequence based on the knowledge of three variables. These are: (1) the extent, pattern and aggressiveness of the initial presentation of metastatic disease; (2) the menopausal status of the patient; (3) the hormone receptor status of the tumour. Hormone treatment has been the preferred initial treatment, chemotherapy being used for patients failing to respond to, or relapsing after, endocrine therapy. Exceptions to this are patients with aggressive visceral disease and those with hormone receptor negative tumours when cytotoxic chemotherapy is the initial treatment of choice (Figure 1).

Additional treatments were used for complications of bone metastases. Hypercalcaemia was initially treated by intravenous rehydration with normal saline. Patients becoming normocalcaemic or remaining only mildly hypercalcaemic commonly received oral phosphates to maintain control. Those remaining hypercalcaemic were, until recently, treated with mithramycin or calcitonin to inhibit bone resorption, but now our present preferred agent for control of hypercalcaemia is 3 amino,l,hydroxypropylidene-l,l-bisphosphonate (APD) (Coleman & Rubens, 1986).

To treat, or to reduce the risk of pathological fracture of a long bone, internal fixation followed by radiotherapy was performed. This usually relieved pain and provided optimum conditions for callus formation and bony union. Spinal cord compression was treated by surgical decompression if the signs were rapidly progressing, but radiotherapy was preferred for slowly progressing, previously non-irradiated sites of compression. Bone marrow infiltration causing leucoerythroblastic anaemia often occurred late in the course of the disease and, when it had become resistant to hormones, treatment was by cautious low dose chemotherapy and haematological support.

### Results

*Incidence and prognosis of bone metastases*

Bone was the most common site of metastatic disease. Four hundred and eighty-five of 587 (69%) patients dying with breast cancer in the 5 year period 1979–84 had radiological evidence of skeletal metastases before death. In comparison 158 (27%) had lung metastases, and 157 (27%) liver metastases.

Bone was also the most common site of first distant relapse. In the past ten years 2240 patients have presented to this Unit with primary breast cancer. Six hundred and eighty-one (30%) have relapsed after a median follow-up of nearly 5 years. Two-hundred and forty-five relapses (36%) were classified as local, 395 (58%) as distant and 41 (6%) concurrent local and distant. One hundred and eighty-four patients relapsed first in bone (47% of all first distant relapse). Table I shows the distribution and relative frequency of first relapse.

The mean age of patients with first relapse in either bone or in liver was 57 years (range 29–79 and 29–78 years in bone and liver respectively). Thirty-one per cent with bone

**Figure 1** Schema for the selection of systemic treatment for advanced breast cancer.

**Table I** Distribution and frequency of first relapse in 2240 patients presenting with primary breast cancer

| *Site* | *Local*[a] | *Bone* | *Distant soft tissue* | *Lung* | *Pleura* | *Liver* | *Brain* | *Other* |
|---|---|---|---|---|---|---|---|---|
| Number of patients | 245 | 184 | 75 | 46 | 30 | 19 | 6 | 35 |
| % total study population | 11 | 8 | 3 | 2 | 1 | 1 | 0.3 | 1 |
| % patients with any relapse (local and distant) | 36 | 24 | 11 | 7 | 4 | 3 | 1 | 4 |
| % patients with distant relapse | — | 47 | 19 | 12 | 8 | 5 | 2 | 9 |

[a]Local relapse defined as recurrence in ipsilateral breast or mastectomy scar, or ipsilateral axillary or supraclavicular lymph nodes.

relapse and 33% with liver relapse were premenopausal. The median disease-free interval before first relapse in either bone or liver was identical at 20 months (range 0–120 for bone and 0–91 months for liver).

The median duration of survival in 498 patients with first relapse in bone was 20 months. In 253 patients with metastatic disease apparently confined to the skeleton the median duration of survival was 24 months. The median duration of survival after first relapse in liver was only 3 months. (Figure 2).

**Figure 2** Survival after first relapse in bone or liver.

First relapse in bone was identified in 498 patients. Two hundred and forty-five (49%) subsequently relapsed at other sites. One hundred and forty-five (29%) developed one or more of the principal complications of bone destruction; hypercalcaemia, pathological fracture or spinal cord compression. (Table II).

*Tumour characteristics*

Table III compares ER and PgR in patients with first relapse in bone and liver metastases (not necessarily first site of relapse). First relapse in bone was more common than the development of liver metastases in ER positive ($P = <0.001$) and PgR positive tumours ($P = <0.05$). Spread to the liver was more likely from ER and/or PgR negative tumours.

Table IV compares the histological grade of the primary tumour in patients with bone metastases and those with

**Table II** Subsequent metastatic spread and complications after first relapse of disease in bone ($n = 498$)

| *Site/complication* | *No.* | % |
|---|---|---|
| Bone only | 253 | 51 |
| Soft tissue | 95 | 19 |
| Liver | 94 | 19 |
| Pleura | 78 | 16 |
| Lung | 72 | 14 |
| Brain | 20 | 4 |
| Hypercalcaemia | 86 | 17 |
| Pathological fracture | 78 | 16 |
| Spinal cord compression | 13 | 3 |

**Table III** Comparison of hormone receptor status of the primary tumour in patients with first relapse in bone and relapse in liver (first or subsequent)

| | *Bone* | | *Liver* | | |
|---|---|---|---|---|---|
| | *No.* | % | *No.* | % | |
| ER+[a] | 120 | (80) | 44 | (59) | $P = <0.001$ |
| ER− | 30 | (20) | 31 | (41) | |
| PgR+ | 66 | (60) | 26 | (43) | $P = <0.05$ |
| PgR− | 44 | (40) | 34 | (57) | |
| ER+PgR+ | 62 | (57) | 20 | (34) | $P = <0.01$ |
| ER+PgR− | 27 | (25) | 15 | (25) | |
| ER−PgR+ | 4 | (4) | 5 | (8) | |
| ER−PgR− | 16 | (15) | 19 | (32) | $P = <0.01$ |

[a]ER+ and PgR+ = >5 fmol mg$^{-1}$ cytosol protein; ER− and PgR− = <5 fmol mg$^{-1}$ cytosol protein.

visceral disease. Bone metastases were more common with well-differentiated tumours. Fifty-seven of 95 (60%) of patients with grade 1 and 120/302 (40%) with grade 3 tumours developed bone metastases ($P = <0.05$). First relapse in bone was also more common from well differentiated tumours. Liver metastases were more common in poorly differentiated tumours but this difference was not significant. The development of either liver or lung metastases (visceral disease) at any time was related to histological grade. Twenty of 95 (21%) of patients with grade 1 and 124/302 (41%) with grade 3 tumours developed visceral disease ($P = <0.001$).

*Response to systemic treatment*

Table V shows the response to primary endocrine treatment of 183 patients with advanced breast cancer. The overall response rate (complete and partial responses) was 35%. One hundred and twelve of 183 patients had bone metastases in

**Table IV** Histological grade of the primary and the distribution of subsequent metastatic relapse

| | *Grade 1* *N* = 95 | | *Grade 2* *N* = 464 | | *Grade 3* *N* = 302 | | | |
|---|---|---|---|---|---|---|---|---|
| | *No.* | % | *No.* | % | *No.* | % | | |
| Bone metastases at any time | 57 | (60) | 223 | (48) | 120 | (40) | 1 *vs.* 2<br>1 *vs.* 3<br>2 *vs.* 3 | $P = <0.05$<br>$P = <0.001$<br>$P = <0.05$ |
| First relapse in bone | 37 | (39) | 155 | (33) | 84 | (28) | 1 *vs.* 2<br>1 *vs.* 3<br>2 *vs.* 3 | NS<br>$P = <0.05$<br>NS |
| Liver metastases at any time | 14 | (13) | 69 | (15) | 66 | (22) | 1 *vs.* 2<br>1 *vs.* 3<br>2 *vs.* 3 | NS<br>NS<br>NS |
| Visceral metastases at any time (liver and/or lung) | 20 | (21) | 129 | (28) | 124 | (41) | 1 *vs.* 2<br>1 *vs.* 3<br>2 *vs.* 3 | NS<br>$P = <0.001$<br>$P = <0.001$ |

**Table V** Overall response and response in bone to primary endocrine therapy

| | *CR* | | *PR* | | *NC* | | *PD* | | *NA* | |
|---|---|---|---|---|---|---|---|---|---|---|
| | *n* | % | *n* | % | *n* | % | *n* | % | *n* | % |
| Overall response (all patients) *n* = 183 | 10 | (5) | 56 | (30) | 42 | (23) | 50 | (27) | 25 | (14) |
| Overall response (bone metastases patients) *n* = 112 | 1 | (1) | 34 | (31) | 31 | (28) | 38 | (34) | 8 | (7) |
| Response in bone *n* = 112 | 0 | | 20 | (18) | 35 | (31) | 30 | (27) | 27 | (24) |

CR = complete response, PR = partial response, NC = no change, PD = progressive disease, NA = not assessable.

whom the overall response rate was 32%, but response in bone only 18%. The rate of progression overall and in bone was similar at 27%. Apparently static disease in bone was common (31%) and 24% of bony sites were unassessable, because of either sclerotic appearance on X-ray, rapid extra-skeletal progression, or lack of follow-up.

Sixty-eight patients had both osseus and non-osseus metastases. Fourteen of 68 (21%) of the former and 27/68 (40%) of the latter showed objective response. The response rate in non-osseus sites was significantly higher ($P = <0.02$).

Similarly response in bone to chemotherapy appeared less common than the overall response. Thirty-seven of 126 (29%) patients, all with bone metastases, treated with combination chemotherapy achieved complete or partial response. Response in bone was less frequent with 18% showing radiological evidence of healing (Table VI). A high proportion of patients (47%) in this series also appeared to have static bone disease.

The survival of patients with bone metastases receiving primary endocrine therapy is shown in Figure 3. Responding patients have the best prognosis with a median duration of survival which exceeds 30 months compared with 7 months in non-responders ($P = <0.001$). There is no significant difference in survival between responding patients and those with static disease although the trend is in favour of those showing objective response. This suggests that a tumour response may occur in the absence of discernable radiological evidence of healing.

### *Morbidity caused by bone metastases*

Hypercalcaemia is a common complication of advanced breast cancer. One hundred and one of 1049 (10%) patients dying with breast cancer in the period 1975–84 developed hypercalcaemia. All patients with hypercalcaemia had

**Table VI** Overall and bone response to chemotherapy (all types[a]) in 126 patients with bone metastases

| | *CR* | *PR* | | *NC* | | *PD* | | *NA* | |
|---|---|---|---|---|---|---|---|---|---|
| | *n* | *n* | % | *n* | % | *n* | % | *n* | % |
| Overall response | 0 | 36 | (29) | 42 | (33) | 28 | (22) | 20 | (16) |
| Bone response | 0 | 23 | (18) | 59 | (47) | 11 | (9) | 33 | (26) |

CR = complete response, PR = partial response, NC = no change, PD = progressive disease, NA = not assessable.

[a]Regimens included adriamycin +/− vincristine, CMF (cyclophosphamide, methotrexate, 5 fluorouracil), mitomycin C + vinblastine, and mitozantrone.

**Figure 3** Survival from the start of primary endocrine treatment in patients with bone metastases ($n=112$) for each response category, partial response (PR), no change (NC) and progressive disease.

**Table VII** Incidence of bone and liver metastases in patients with hypercalcaemia and breast cancer ($n=147$)

| *Skeletal involvement* | | | | | | | |
|---|---|---|---|---|---|---|---|
| *Widespread* | | | | *Limited* | | | |
| *Total* | | *With liver metastases* | | *Total* | | *With liver metastases* | |
| *No.* | % | *No.* | % | *No.* | % | *No.* | % |
| 125 | 85 | 53 | 42 | 22 | 15 | 15 | 68 |

metastatic disease. One hundred and twenty-five of 147 (85%) patients with hypercalcaemia had evidence of multiple bone metastases. Twenty-two of 147 (15%) had minimal or no evidence of skeletal involvement identified by either radionuclide bone scan or X-ray.

Liver metastases were identified before or at the time of hypercalcaemia in 68/147 patients (46%). Liver involvement was more common in patients with little or no skeletal involvement ($P<0.05$) (Table VII). Eighty-six of 498 (17%) of patients with first relapse in bone developed hypercalcaemia. In those with metastatic disease confined to the skeleton 15% subsequently became hypercalcaemic, compared with 31% of 94 patients who had additional spread to the liver ($P<0.001$). Patients with hypercalcaemia have a poor prognosis despite active treatment. The median survival after hypercalcaemia is 3 months (range 0–56 months) and was similar in patients with or without liver metastases.

Pathological fracture of a long bone occurred in 78/498 (16%) patients after first relapse in bone. The median duration of survival after fracture was 12 months (range 0–66 months). Thirteen of 498 developed spinal cord compression with a median survival of 3 months (range 0–26 months) after this complication.

### Discussion

Bone metastases are common in advanced breast cancer and the 69% incidence of radiologically confirmed skeletal metastases here is in accordance with the 47–85% incidence reported in autopsy series (Gelasko, 1981). Lung or liver metastases were each identified before death in 27% of women dying with breast cancer. This however is less than the 57–77% and 50–71% incidence of disease in lung and liver respectively reported in autopsy series (Di Pietro *et al.*, 1976) indicating relatively frequent asymptomatic disease at these sites as well as reflecting the insensitivity of clinical and imaging techniques for detecting disease in these organs.

Bone is also the commonest site of first distant relapse of breast cancer (McNeil, 1984). In this study bone was the first site of distant relapse in 47% and viscera (liver, lung, pleura) in 25%. Despite the relative frequency of bone relapse this occurred in only 8% of 2240 patients presenting to this Unit with primary breast cancer. This low incidence in an unselected population helps explain why routine bone scanning in the follow-up of early breast cancer is not cost effective (Lee, 1985).

Age and disease free interval were similar in patients with first relapse in either bone or in liver. Relapse in liver was not more common in pre-menopausal patients despite the tendency for lower receptor values in this group (Croton *et al.*, 1981). Metastatic bone disease, unlike liver disease, frequently followed a protracted clinical course. The median duration of survival was 24 months in patients with disease apparently confined to the skeleton.

This study confirms that bone metastases are more common in well differentiated receptor positive tumours, while liver metastases are more frequent with receptor negative anaplastic tumours (Elston *et al.*, 1980).

Clinical trials in the treatment of breast cancer using the UICC criteria of response have usually reported lower reponse rates for bone lesions than the overall response (Stewart *et al.*, 1982). Possibly this could be a true phenomenon if bone metastases are biologically different and relatively refractory to treatment, but it seems more likely that the difference is apparent rather than real because of the insensitivity of assessment methods. Indeed the association between bone metastases and receptor positive, well differentiated tumours would predict a high response rate.

This underestimation of response to both primary endocrine therapy and chemotherapy was also seen in this study. The incidence of progressive disease was similar, but the proportion of patients with non-assessable or apparently stable disease was higher in bone. Survival from the start of endocrine treatment was similar in patients with either objective response or stable disease.

We are currently evaluating alternative response criteria including radionuclide bone scanning, biochemical parameters of bone metabolism and subjective assessment. Preliminary results show that prediction of objective response within 3–4 weeks of starting treatment is possible (Coleman *et al.*, unpublished data).

Morbidity from bone metastases was common. Twenty-nine percent of patients with first relapse in bone subsequently developed one or more of the major complications of bone destruction, hypercalcaemia, pathological fracture and spinal cord compression. The prognosis of patients with hypercalcaemia was poor with a median duration of survival of 3 months but prolonged remissions up to 56 months are seen. Better prediction of impending pathological fracture and early orthopaedic intervention might reduce the frequency of this unpleasant complication.

This review of a single Unit's large experience of patients with bone metastases treated by a logical sequence of treatments has presented data on various clinical and pathological aspects of metastatic bone disease. It has confirmed the clinical importance of bone metastases, the long and usually symptomatic clinical course, the relationship between tumour characteristic and sites of metastatic disease, and the difficulties in assessing response to systemic therapy. New approaches to treatment and more accurate methods of assessment are needed to improve management.

We wish to thank Prof. M.N. Maisey and Dr I. Fogelman for radionuclide investigations, Dr R.J.B. King for steroid receptor analyses, Dr R. Millis for histology and grading of tumours and Mr S. Edmeades for assistance in data handling.

### References

BLOOM, H.J.G. & RICHARDSON, W.W. (1957). Histological grading and prognosis in breast cancer: A study of 1409 cases of which 359 have been followed for 15 years. *Br. J. Cancer*, **11**, 359.

COLEMAN, R.E. & RUBENS, R.D. (1986). Treatment of hypercalcaemia secondary to metastatic breast cancer with 3-amino-i-hydroxypropylidene-l,l-bisphosphonate (APD). *Br. J. Cancer*, **54**, 203 (*abstract*).

COLEMAN, R.E. & RUBENS, R.D. (1985). Bone metastases and breast cancer. *Cancer Treatment Rev.*, **12**, 251.

CROTON, R., COOKE, T., HOLT, S., GEORGE, W.D., NICOLSON, R. & GRIFFITHS, K. (1981). Oestrogen receptors and survival in early breast cancer. *Br. Med. J.*, **283**, 1289.

DI PIETRO S., BERTARIO, L., CARTA, G. & RE, A. (1976). An analysis of 800 breast cancer patients relapsed after radical mastectomy. *Tumori*, **62**, 99.

ELSTON, C.W., BLAMEY, R.W. & JOHNSON, J. (1980). The relationship of oestradiol receptor (ER) and histological tumour differentiation with prognosis in human primary breast carcinoma. In *Breast Cancer: Experimental and Clinical Aspects*, Mouridsen, H.T. & Palshof, T. (eds) p. 59. Pergamon: Oxford.

GALASKO, C.S.B. (1981). The anatomy and pathways of skeletal metastases. In *Bone Metastasis*, Weiss, L. & Gilbert, H.A. (eds) p. 49. G.K. Hall: Boston, Massachusetts.

HAYWARD, J.L., CARBONE, P.P., HEUSON, J.C., HUMAOKA, S., SEGALOFF, A. & RUBENS, R.D. (1977). Assessment of response to therapy in advanced breast cancer. *Eur. J. Cancer*, **13**, 89.

KING, R.J.B., REDGRAVE, R., HAYWARD, J.L., MILLIS, R.R. & RUBENS, R.D. (1979). The measurement of receptors for oestradiol and progesterone in human breast tumours. In *Steroid Receptor Assays in Breast Tumours; Methodological and Clinical Aspects*, King, R.J.B. (ed) p. 55. Alpha Omega: Cardiff.

LEE, Y.-T.M. (1985). Patterns of metastasis and natural causes of breast carcinoma. *Cancer Metastasis Rev.*, **4**, 153.

McNEIL, B.J. (1984). Value of bone scanning in neoplastic disease. *Sem. Nuclear Med.*, **14**, 277.

STEWART, J., KING, R., HAYWARD, J. & RUBENS, R.D. (1982). Estrogen and progesterone receptors: Correlation of response rates, site and timing of receptor analysis. *Breast Cancer Res. Treatment*, **2**, 243.